# Orthogonal Chirp Division Multiplexing for Underwater Acoustic Communication

**DOI:** 10.3390/s18113815

**Published:** 2018-11-07

**Authors:** Yiqi Bai, Pierre-Jean Bouvet

**Affiliations:** 1 College of Information Science and Technology, Ocean University of China, Qingdao 266100, China; byq@stu.ouc.edu.cn; 2 SEACom Department, L@bisen Yncréa-Ouest, 29228 Brest CEDEX 2, France

**Keywords:** UWAC, OFDM, OCDM

## Abstract

The objective of this study is to investigate a novel Underwater Acoustic Communication (UWAC) system based on a modulated chirp signal termed as Orthogonal Chirp Division Multiplexing (OCDM). Originating from the Fresnel transform, OCDM uses chirp signals to exploit the multipath diversity of the channel, achieving a good robustness against frequency fading, especially in the underloaded scenario where only a subset of the available waveforms is modulated. The implementation of the OCDM system for the UWAC scenario is described, and the performance results over an experimental water tank and realistic replayed underwater channel are compared against the traditional Orthogonal Frequency Division Multiplexing (OFDM) transmission scheme.

## 1. Introduction

UWAC has multiple applications ranging from military to commercial fields, including scientific uses. However, the severe characteristics of the underwater acoustic channel such as time variation, high ambient noise, limited bandwidth and extended multipath effect make data transmission through underwater acoustic signals very challenging [1,2,3,4]. By the use of frequency sweep signals, which are resistant to the detrimental effects of the UWAC channel, the Chirp Spread Spectrum (CSS) modulation technique offers robust performance with a very simple matched filtering-based decoder that makes such a communication scheme particularly adapted to the UWAC channel [5,6]. In the CSS system, a broad spectrum is occupied to modulate the information in order to achieve high processing gain and multipath resolution to the detriment of the spectral efficiency. As a result, CSS is very attractive for a low and medium data rate UWAC link when reliability is considered as a priority factor. In order to optimize data rates with respect to the occupied bandwidth, research works in the 1990s focused on Single Carrier (SC) coherent Phase Shift Keying (PSK) and Quadrature Amplitude Modulation (QAM) signaling for UWAC. These modulation methods offer high spectral efficiencies, but require complex receivers that need to equalize extensive Channel Impulse Response(CIR) while tracking time variations and phase distortions. Nowadays, turbo-equalization approaches in the time [7,8,9] or frequency domain [9,10] are often preferred for UWAC since these algorithms provide a good trade-off between performance and complexity.

On the other hand, in the last decade, the conversion of OFDM modulation [11] to UWAC has been deeply investigated in the literature in order to improve the data rate of underwater telemetry while keeping a one tap equalizer at the reception side. OFDM-based UWAC modulation systems have demonstrated high spectral efficiencies in real undersea experiments, but also sensitivity to Doppler spread that breaks the subcarriers orthogonality, yielding to Inter-Carrier Interference (ICI). Thus, UWAC systems based on OFDM often require advanced ICI compensation algorithms [12,13].

Very recently, a new modulation scheme called OCDM has been introduced in the field of optical fiber communications [14]. The basic principle of OCDM consists of multiplexing a number of chirp waveforms that are mutually orthogonal with each other and sharing the same bandwidth and time slot. With respect to single chirp modulation, OCDM increases the transmitted data rate by overlapping data in both the time and chirp domain. On the other hand, the OCDM approach shares many similarities with the OFDM system like orthogonal waveform multiplexing, which is based on the Fresnel transform [15], instead of the Fourier transform, as in OFDM. In a previous paper [16], the use of OCDM for UWAC was experimented with, yielding very promising results, but the study was limited to a simulated UWAC channel. The purpose of this paper is to extend the results of [16] to more realistic transmission channels in order to illustrate the feasibility of the OCDM system in real scenarios and its advantages against the conventional OFDM system.

This paper is organized as follows: Section 2 describes the OCDM concept and its formulation. In Section 3, the detailed transmission structure is presented, which includes the modulator and receiver algorithms and the experimental channel. Section 4 provides the experimental performance over a static tank channel and replayed dynamic experimental channels, where OCDM is demonstrated to outperform conventional OFDM under certain circumstances. Finally, Section 5 presents the conclusion of this paper.

Notation: ${\left(\cdot \right)}^{*}$ stands for conjugate, ${\left(\cdot \right)}^{T}$ for transpose and ${\left(\cdot \right)}^{H}$ for Hermitian transpose. x denotes a column vector, and X is a matrix. Matrix $I_{N}$ denotes a N×N identity matrix and $0_{1\times N}$ stands for the 1×N all-zeros vector. Moreover, ${\left\{X\right\}}_{i,j}$ denotes the *i*-th row of the *j*-th column of X. Finally, E[.] denotes the statistical expectation.

## 2. OCDM Basics

### 2.1. Principle of OCDM

Originating from the Fresnel transform [15], a set of orthogonal chirp signals has been proposed in [14] as the kernel of OCDM. Let $\psi_{n}\left(t\right)$ be a set of waveforms with chirp wave-number $n\in \left[1,N\right]$ as follows:

(1) $$\psi_{n}\left(t\right)=e^{j\frac{\pi }{4}}e^{-j\pi \frac{N}{T^{2}}{\left(t-n\frac{T}{N}\right)}^{2}},t\in \left[0,T\right]$$

**Figure 1:**
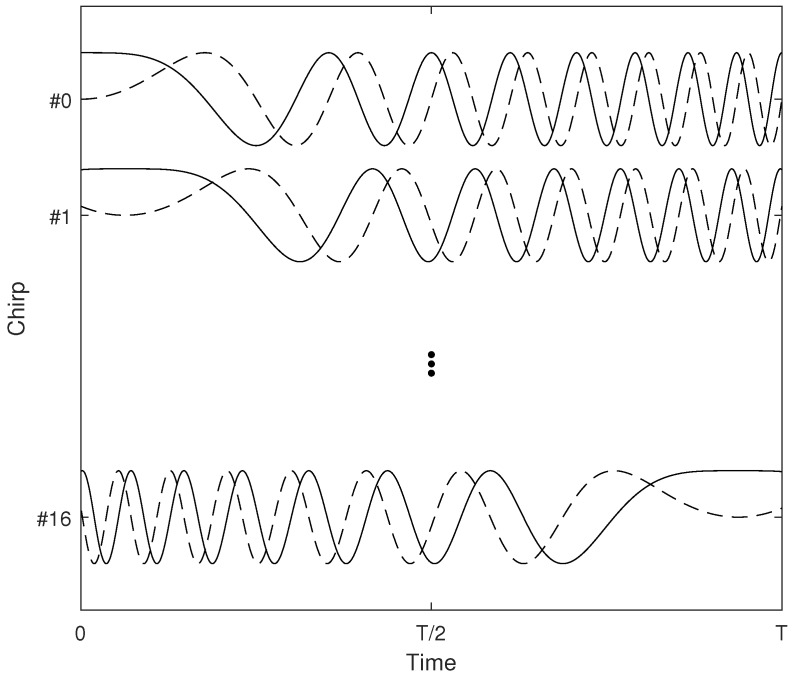
OCDM chirp waveforms for N=16.

As an example, a 16-chirped OCDM system is illustrated in Figure 1, where the real part of chirped waveforms is depicted in a solid line, whereas the dashed line stands for the imaginary part. It it obvious that these chirp waveforms are mutually orthogonal in the chirp dimension:

(2) $$\int_{0}^{T}\psi_{m}^{*}\left(t\right)\psi_{n}\left(t\right)dt=\int_{0}^{T}e^{j\pi \frac{N}{T^{2}}{\left(t-m\frac{T}{N}\right)}^{2}}e^{-j\pi \frac{N}{T^{2}}{\left(t-n\frac{T}{N}\right)}^{2}}dt=\left\{\begin{array}{cc} 1 & \mathrm{if}\;m=n\\ 0 & \mathrm{instead} \end{array}\right.$$

In the time domain, the useful baseband transmission signal for the OCDM block *k* labeled as $s_{k}\left(t\right)$ can be obtained by multiplexing these *N* waveforms over the duration *T* where each waveform is multiplied by a QAM cell $x_{k}^{n}$ carrying the useful information:

(3) $$s_{k}\left(t\right)=\sum_{n=0}^{N-1}x_{n}^{k}\psi_{n}\left(t\right),t\in \left[0,T\right]$$

Similar to OFDM [13], the orthogonality property of (2) can be exploited to recover data in the receiving block $r_{k}\left(t\right)$:

(4) $${\hat{x}}_{n}^{k}=\int_{0}^{T}r_{k}\left(t\right)\psi_{n}^{*}\left(t\right)dt$$

### 2.2. OCDM in Matrix Form

Equations (3) and (4) realize the basic concept of OCDM for analog signals. For a digital signal, OCDM modulation is performed by using Inverse Discrete Fresnel Transform (IDFnT), as shown in [14]. In fact, sampling (3) at mT/N yields:

(5) $$\begin{array}{c} s_{m}^{k}=s_{k}{\left(t\right)}_{t=m\frac{T}{N}}=\sum_{n=0}^{N-1}x_{n}^{k}\psi_{n}\left(mT/N\right)=e^{j\frac{\pi }{4}}\sum_{n=0}^{N-1}x_{n}^{k}e^{-j\frac{\pi }{N}\left(m-n\right.{)}^{2}} \end{array}$$

From the above expression, the concise matrix form of OCDM modulation can be expressed as:

(6) $$s_{k}=\Phi^{H}x_{k}$$

with $s_{k}={\left[\begin{array}{ccc} s_{0}^{k} & \ldots & s_{N-1}^{k} \end{array}\right]}^{T}$, $x_{k}={\left[\begin{array}{ccc} x_{0}^{k} & \ldots & x_{N-1}^{k} \end{array}\right]}^{T}$ and unitary matrix Φ, which is the so-called Discrete Fresnel Transform (DFnT) matrix defined as follows for an even number *N*:

(7) $${\left\{\Phi \right\}}_{m,n}=\frac{1}{\sqrt{N}}e^{-j\frac{\pi }{4}}e^{j\frac{\pi }{N}\left(m-n\right.{)}^{2}}$$

In the demodulation stage, a DFnT operation is invoked as follows:

(8) $${\hat{x}}_{k}=\Phi s_{k}$$

### 2.3. Underloaded OCDM System

The concept of underloaded OCDM consists of modulating a useful number $N_{u}$ of chirp waveforms that is inferior to the total number *N* of available waveforms. Under this situation, the vector of information data $x_{k}$ in (6) can be expressed as:

(9) $$x_{k}=\sqrt{\frac{N}{N_{u}}}{\left[\begin{array}{ccccc} x_{k}^{0} & 0_{1\times N/N_{u}-1} & \cdots & x_{k}^{N_{u}-1} & 0_{1\times N/N_{u}-1} \end{array}\right]}^{T}$$

For computation simplicity, $N_{u}$ is set to an integer divisor of *N*; for example, $N_{u}=\frac{1}{2}N$ indicates that half of the total waveforms are being used. Note that the coefficient $\sqrt{N/N_{u}}$ is introduced in order to keep the same signal energy whatever $N_{u}$. As depicted in Section 4, the underloaded approach leads to Signal-to-Noise Ratio (SNR) gain, but at the expense of the decreasing data rate. The particular case when $N_{u}=1$ corresponds to CSS modulation. The same underloaded approach can also be applied to OFDM systems.

## 3. System Model

### 3.1. Transmission Structure

This section describes the OCDM transmission system. Information data $d_{n}$ are first encoded by Forward Error Correcting (FEC) code and then randomly interleaved along the frame and mapped to the QAM constellation. The chosen FEC coder listed above is a 64-state convolutional encoder with polynomial generator ${\left(156,123\right)}_{o}$ and rate $R_{c}=1/2$. These combined coding methods are introduced in order to reach high communication efficiency, good accuracy performance and error correction capability against the UWAC channel.

**Figure 2:**
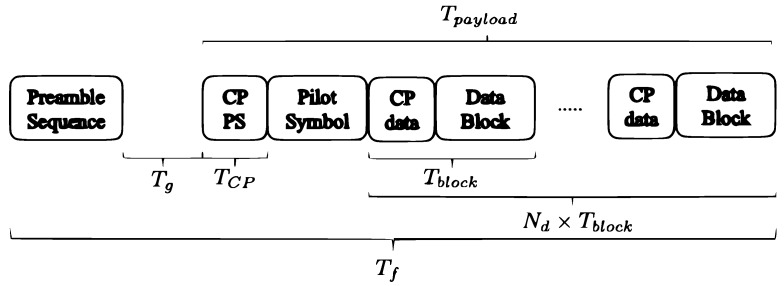
Frame structure.

The OCDM modulation is performed on a coded data block and then rearranged according to the framing structure described in Figure 2: each frame includes one Pseudo-Noise (PN) preamble sequence to perform frame synchronization and Doppler estimation, one pilot symbol for channel estimation and $N_{d}$ data blocks. In order to avoid Inter-Symbol Interference (ISI), a Cyclic Prefix (CP) is inserted between adjacent blocks. The CP length $N_{CP}=NR_{CP}$ is set according to the maximum channel delay time. Power normalization is applied such that the variance of transmitted symbols is unitary, i.e., $E[|x_{n}^{k}{|}^{2}]=1$.

### 3.2. Experimental Channels

Two channels are considered in this paper, a tank channel for static performance tests and a replay channel reproducing real-world at-sea acoustic transmission. In both experiments, the CP duration is set much longer than the Root Mean Square (RMS) channel delay spread [17] in order to absorb most of the multipath energy.

#### 3.2.1. Water Tank

**Figure 3:**
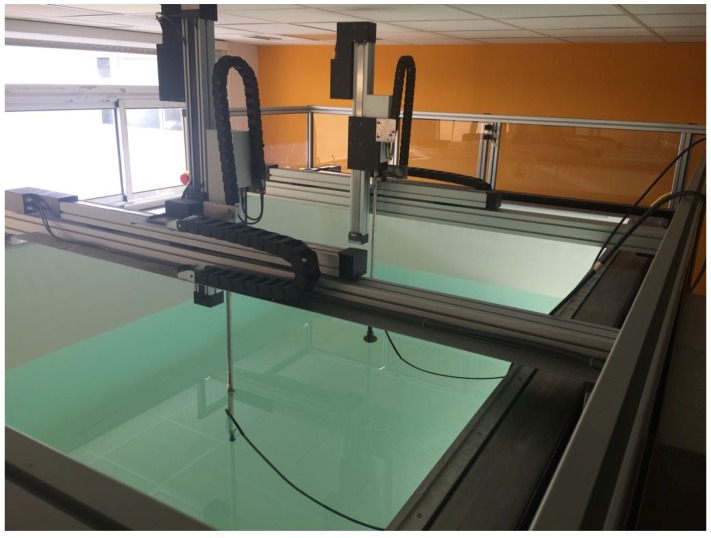
Experimental water tank.

**Figure 4:**
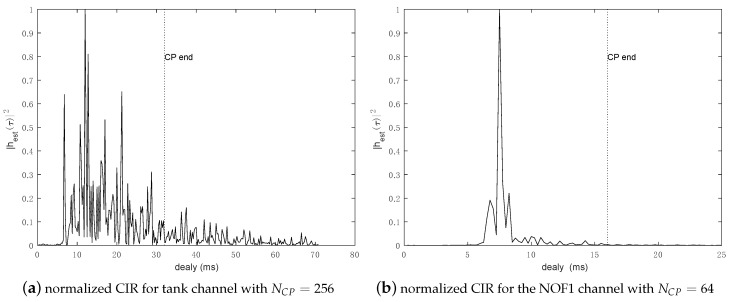
Channel impulse response for tank and NOF1 channels, the cyclic prefix tolerance as the dotted line.

The static channel experiment is based on an experimental water tank (2 m × 1 m × 1 m) situated on the L@bisen Yncréa-Ouest (Brest, France) premises and depicted in Figure 3. At the transmission side, a Neptune Sonar Limited (Kelk, United Kingdom) D/26 is used, whereas at the reception side, we use a Brüel & Kjaer (Nærum, Denmark) 8104 hydrophone. The range between the projector and the hydrophone is 1 m. Channel parameters are summarized in Table 1, and estimated CIR is depicted in Figure 4a. Large channel delay spread is caused by multiple reflections on tank boundaries. Furthermore, due to the tank configuration, CIR is assumed static during the experimentation.

#### 3.2.2. Watermark NOF1 Channel

In order to simulate a real experiment, we consider the Watermarkchannel [18] that implements a replayed channel simulator driven by at-sea measurements of the time-varying CIR. The Watermark channel is a realistic simulation tool including a direct-replay channel simulator and test channel document freely available to the UWAC community. The simulation tool includes Single-Input Single-Output (SISO) and Single-Input Multiple-Output (SIMO) modes. The principle of the simulator consists of distorting input waveforms by convolving them with measured channels [19]. Such an operation known as channel replay can be expressed as:

(10) $$r\left(t\right)=\int_{-\infty }^{\infty }\hat{h}\left(t;\tau \right)s\left(t-\tau \right)d\tau +n\left(t\right)$$

where $s\left(t\right)$ is the input signal, $\hat{h}\left(t;\tau \right)$ the recorded Time-Varying Impulse Response (TVIR) estimates, $n\left(t\right)$ a noise term and $r\left(t\right)$ the distorted output signal. On the other hand, a channel file is represented by aMATLAB (.mat) file with a continuous single-hydrophone TVIR estimate. The collection represents consecutive soundings (TVIR measurements). To reproduce at-sea conditions, Watermark also includes Doppler shifts due to both platform motion and Carrier Frequency Offset (CFO).

The selected Watermark channel for this paper is NOF1 (Norway–OsloFjord) with a signaling range of 750 m and a water depth of 10 m. The channel coherence time for a normalized CIR autocorrelation value of 0.75 is found to be 2.56 s. The NOF1 CIR estimated from the preamble sequence is shown in Figure 4b, and the basic parameters are listed in Table 1.

### 3.3. Decoding Structure

**Figure 5:**
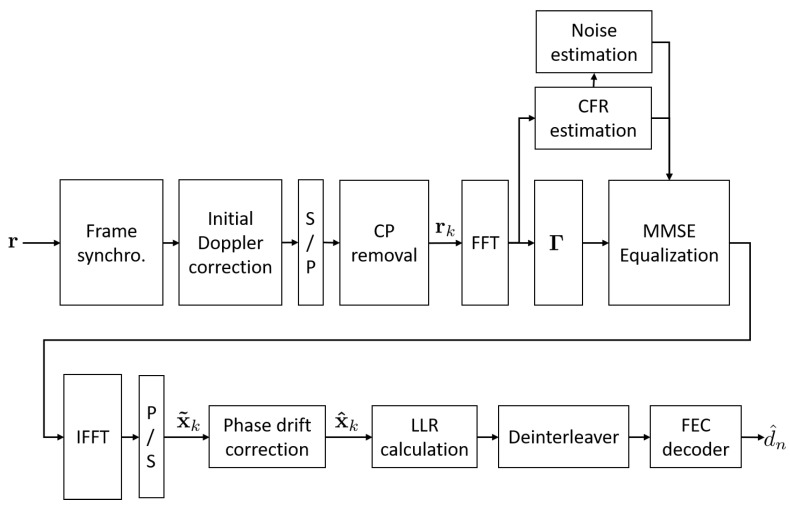
Decoder structure.

The decoding process structure considered in this study is mainly based on the decoder architecture introduced in [14] from which the UWAC Doppler processing and channel decoding block are added. The overall schematic flowchart of the decoder is illustrated in Figure 5. After base-band conversion, frame synchronization is performed by cross-correlation between the received stream and the PN sequence, then motion-induced Doppler shifts are estimated and compensated by resampling and phase correction [12]. After serial-to-parallel conversion and CP removal, for each block *k*, a vector noted $r_{k}$ is obtained. In order to remove the multipath effect brought by the UWAC channel, Frequency Domain Equalization (FDE) is invoked by applying Fast Fourier Transform (FFT) to $r_{k}$, leading to the vector $y_{k}$, which can be expressed as [14]:

(11) $$y_{k}=F\cdot r_{k}=\Gamma^{H}\Lambda Fx_{k}+w_{k}$$

where F is the Fourier matrix of size *N*, Γ and Λ are both diagonal matrices of size N×N with:

(12) $${\left\{\Gamma \right\}}_{n,n}=e^{-j\frac{\pi }{N}n^{2}}$$

in the case of *N* being even and:

(13) $${\left\{\Lambda \right\}}_{n,n}=H_{n}$$

where $H_{n}$ is the Channel Frequency Response (CFR) of the channel at the *n*-th frequency bin. Additionally, $w_{k}$ is the resulting noise vector assumed Gaussian with zero-mean and variance noted $\sigma_{w}^{2}$.

Under the assumption that the CFR is constant during the frame, an estimation of the transmitted vector ${\tilde{x}}_{k}$ can be formed by compensating the CFR and the phase rotation induced by matrix Γ and then transferred to the time domain via inverse FFT as follows:

(14) $${\tilde{x}}_{k}=F^{H}G\Gamma y_{k}$$

where G is a diagonal equalization matrix optimized under the Minimum Mean Square Error (MMSE) criterion such that:

(15) $$G=\Lambda^{H}{\left(\Lambda^{H}\Lambda +\sigma_{w}^{2}I\right)}^{-1}$$

In practice, the equalization matrix is computed from an estimation of CFR obtained from the pilot symbol with the conventional Least Square (LS) algorithm.

Then, in order to compensate the residual Doppler shift within the equalized signal, a fine phase correction stage is applied as:

(16) $${\hat{x}}_{n}^{k}=e^{-j{\hat{\phi }}_{d}}{\tilde{x}}_{n}^{k}$$

The estimation of the Doppler phase shift ${\hat{\phi }}_{d}$ is initialized to zero at the beginning of each frame and updated for each OCDM block by measuring the average phase rotation between previous estimates and the closest constellation points:

(17) $${\hat{\phi }}_{d}={\hat{\phi }}_{d}-\frac{1}{N}\sum_{n=1}^{N}\arg\left({\left({\hat{x}}_{n}^{k-1}\right)}^{*}\cdot \mathrm{Dec}\left({\tilde{x}}_{n}^{k-1}\right)\right)$$

where Dec(.) refers to the decision process choosing the closest complex cell from the QAM constellation. Finally, from estimated QAM data, Logarithm Likelihood Ratio (LLR) is formed for each bit, then the data are de-interleaved and finally fed to a Viterbi-based channel decoder providing an estimation of the data bits.

When considering system complexity, it can be shown that IDFnT, in the transmission side, can be efficiently performed by using an Inverse Fast Fourier Transform (IFFT) yielding a complexity increase of only two phase multiplications per symbol with respect to traditional OFDM [14]. At the reception side, an additional IFFT is required compared to OFDM that brings an additional complexity of $0.5\log_{2}N$ per chirp/sub-carrier. By combining the transmitter and the receiver, the total complexity increase is $2+0.5\log_{2}N$ multiplications per symbol, representing a slight increase with respect to OFDM.

## 4. Results

### 4.1. System Parameters

In this section, the performance of OCDM is compared against the conventional OFDM system over static and dynamic UWAC channels. The evaluation will be based on three performance metrics: Mean Square Error (MSE) between $x_{n}^{k}$ and ${\hat{x}}_{n}^{k}$, Bit Error Rate (BER) after Viterbi decoding and Effective Data Rate (EDR), which is defined as:

(18) $$\mathrm{EDR}=\left(1-\mathrm{PER}\right)\cdot D_{r}$$

where the Packet Error Ratio (PER) denotes the percentage of the erroneous frame w.r.t. the total transmitted frames (a frame is erroneous if one bit is not properly decoded), and the raw data rate $D_{r}$ is defined as:

(19) $$D_{r}=\frac{N_{d}N_{u}}{N\left(1+N_{d}\right)\left(1+R_{CP}\right)}BR_{c}\log_{2}M\;\mathrm{bps}$$

The system parameters for both the water tank and Watermark NOF1 replayed channel experiments are summarized in Table 2. Some of these parameters are shared by both OCDM and OFDM systems so as to provide a fair comparison.

### 4.2. Water Tank Channel

**Figure 6:**
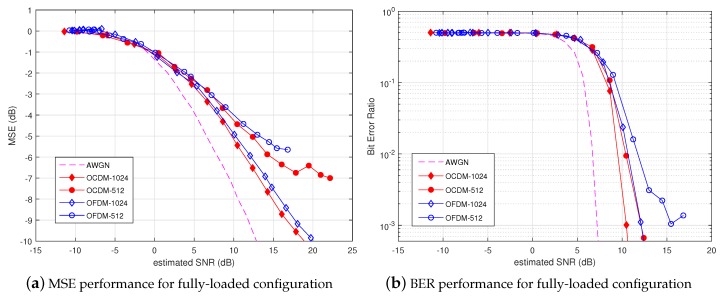
Performance comparison of fully-loaded OCDM and OFDM over the water tank channel.

The tank experiment allowed comparing the performance of OCDM against conventional OFDM in a static scenario, meaning that neither Doppler shift, nor Doppler spread effects were observed in the received signal. However, multiple reflections on the surface, bottom and tank edges provided an extensive multipath effect. In order to avoid ISI, the CP length was set such that $N_{CP}/B$ was much larger than the RMS channel delay spread. The performance of MSE and BER is plotted respectively in Figure 6a,b. Moreover, the performance in Additive White Gaussian Noise (AWGN) channel is drawn in both figures to provide a lower bound on performance. It can be concluded that fully-loaded OCDM provided a slight MSE enhancement compared with fully-loaded OFDM on the static channel. This MSE gain was logically converted to BER enhancement, as shown in Figure 6b. One can note that the BER gain was getting higher as *N* decreased. In fact, since $R_{CP}$ is constant, by decreasing *N*, the absolute CP duration was getting shorter. This phenomenon demonstrates the better robustness of OCDM against out of guard interval echoes. Furthermore, the performance gain can be explained by the better diversity exploitation provided by the OCDM approach where each information cell was spread over a full block duration and frequency band contrary to OFDM, which carried the information cell on a given sub-carrier. Besides, robustness gain would be larger without FEC encoding and random interleaving, which provided frequency and time diversities [14].

**Figure 7:**
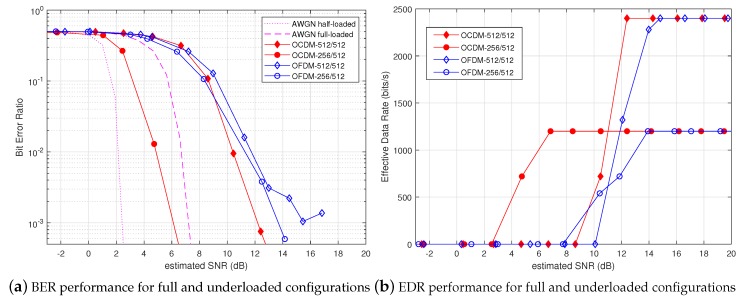
Performance comparison of the full and underloaded OCDM and OFDM over the water tank channel.

Results were substantially different in the underloaded scenario presented in Figure 7a. When $N_{u}/N=1/2$, OCDM achieved huge improvement w.r.t. OFDM, especially in the low SNR region. This performance gain was due to the spreading spectrum effect realized by OCDM in the underloaded configuration. The same behavior can be seen in the EDR curves depicted in Figure 7b, where the half-loaded OCDM modulation achieved a steady EDR of 1.2 kb/s from an SNR of 7 dB, which represents an impressive performance gain w.r.t. half-/fully-loaded OFDM, as well as fully-loaded OCDM. Actually, underloaded OCDM was demonstrated to combine diversity and spreading gain efficiently, therefore leading to good robustness at low SNR.

### 4.3. Watermark NOF1 Channel

In this section, the UWAC systems with OCDM and OFDM are compared over the Watermark NOF1 channel that replays a real experiment. As shown in Section 3, the time coherence of the NOF1 channel was much smaller than that of water tank channel, leading to non-negligible Doppler shift and Doppler spread effects in the received stream.

As a benchmark, we also provided the performance of an SC transmission system associated with an iterative FDE [9,20]. By performing joint equalization and FEC decoding, the iterative receiver provided excellent performance against ISI and high spectral efficiency, while keeping low arithmetic complexity at the receiver side, making such an approach a reference for UWAC systems [9]. The choice of FDE instead of Time Domain Equalization (TDE) in the iterative loop [7] made the comparison with the proposed OCDM systems as fair as possible. The SC-FDE transmission system can use the same frame structure depicted in Figure 2, leading to a similar data rate and bandwidth. In this article, we have implemented a feed-forward linear equalizer associated with a feedback interference canceler, both optimized under the MMSE criterion (see [9] for details). For the considered channel, six iterations were required for the turbo-equalization process to converge.

**Figure 8:**
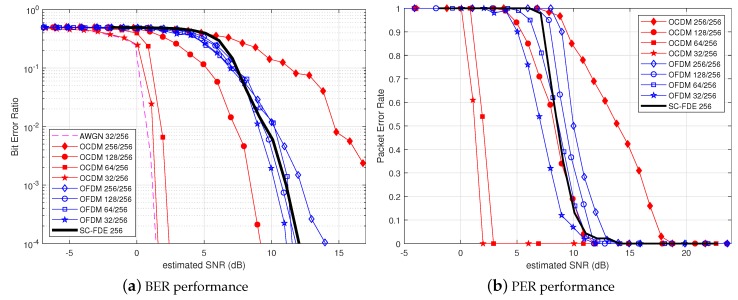

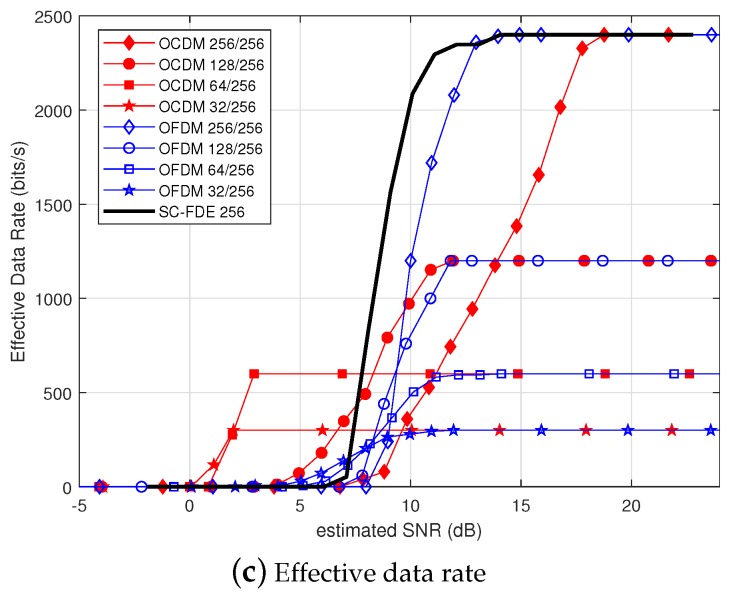
System performance comparison of OCDM, OFDM and SC-FDE over the Watermark NOF1 channel.

Figure 8a,b provides the BER and PER performances of the OCDM, OFDM and SC-FDE systems. In the case of the fully-loaded scenario, conventional OFDM outperformed OCDM. This phenomenon can be explained by noise enhancement induced by the MMSE equalizer in the case of Doppler spread, which cannot be efficiently balanced by the better diversity exploitation offered by OCDM. However, we can see that the SC-FDE system provided higher robustness than fully-loaded OFDM. On the one hand, the turbo-equalization process removed ISI terms induced by the multipath channel efficiently, and on the other hand, SC transmission was less sensitive to CFO due to Doppler spread effects than OFDM waveforms for which CFO larger than inter-carrier spacing resulted in ICI [13]. However, compared to the OCDM and OFDM decoders, the iterative receiver used for the SC transmission system had a higher complexity since several loops of equalization and channel decoding processes were required [20].

In the half-loaded case, OCDM achieved a significant enhancement in the relatively low SNR region, reaching a BER of $10^{-4}$ at an SNR =10 dB, resulting in a 3-dB gain w.r.t. conventional OFDM at the same load. By reducing the load ratio, OCDM still provided BER and PER improvements and converged to the AWGN boundary, while the underloaded performance OFDM of remained slightly improved. This phenomenon is explained by the fact that underloaded OCDM provided both spreading and diversity gain, whereas underloaded OFDM provided only better robustness against CFO due to the inter-carrier spacing increase. As a result, SC-FDE and underloaded OFDM have comparable BER performance.

When analyzing the EDR of the considered systems, SC-FDE outperformed both full-load OCDM and OFDM for high SNR. In fact, SC-FDE was relatively robust against the UWAC channel without sacrificing spectral efficiency. However, at low SNR, the proposed underloaded OCDM provided higher EDR than other studied systems, demonstrating the interest in the this approach for robust UWAC applications. Furthermore, the performance of SC-FDE may be moderated by the fact that the OCDM (and OFDM) system considered in this study could be improved by a turbo-equalization process similar to that employed in SC-FDE.

## 5. Conclusions

The aim of this paper is to evaluate the feasibility of OCDM with a basic principle originating from the Fresnel transform that uses a set of orthogonal chirp waveforms to transmit information data instead of sine waves as OFDM. The transmission and reception structures of OCDM are relatively close to traditional OFDM ones, except for the decoding stage, which requires an FDE algorithm. By evaluating the MSE, BER and EDR performances, OCDM is demonstrated to achieve good spectral efficiencies, while providing high robustness against the UWAC channel by efficiently exploiting the time and frequency diversities of the channel. Performance comparisons against conventional OFDM and SC systems over different UWAC channels prove that in the fully-loaded system, the enhancement of OCDM is not obvious. However, by modulating only a subset $N_{u}$ of the *N* orthogonal waveforms, OCDM provides both spreading and diversity gains, leading to quasi-error-free transmission at low SNR. In such underloaded configurations, the OCDM system is demonstrated to be clearly superior to traditional OFDM and also outperforms the SC system with iterative FDE, making OCDM an interesting technique for robust UWAC applications.

## Figures and Tables

**Table 1:** Channel parameters.

Parameters	Water Tank Channel	Watermark NOF1 Channel [18]
Center frequency	23 kHz	14 kHz
Signal bandwidth	6 kHz	8 kHz
Transmission range	1 m	750 m
Water depth	50 cm	10 m
RMS channel delay spread [17]	16 ms	3.3 ms

**Table 2:** System parameters.

Symbol	Signification	Water Tank Channel	Watermark NOF1 Channel
*B*	Bandwidth for baseband signal	4 kHz	4 kHz
$f_{s}$	Sample frequency in passband	120 kHz	120 kHz
$f_{c}$	Center carrier frequency	20 kHz	14 kHz
$g_{f}$	FEC coder	${\left(156,123\right)}_{o}$	${\left(156,123\right)}_{o}$
$R_{c}$	FEC rate	1/2	1/2
*M*	Constellation order	4	4
$R_{CP}$	Cyclic prefix ratio	1/4	1/4
*N*	Number of waveforms	512 or 1024	256
$N_{u}/N$	Useful waveforms ratio	1/2 or 1	1/8, 1/4, 1/2 or 1
$N_{d}$	Number of data blocks per Frame	3	3
$T_{g}$	Guard interval time	256 ms	256 ms
$N_{ps}$	Preamble Sequence length	512 or 1024	256
$T_{f}$	Frame length	960 or 1472 ms	640 ms
$D_{r}$	Raw data rate	1.2 or 2.4 kb/s	0.3, 0.6, 1.2 or 2.4 kb/s

## Abbreviations

The following abbreviations are used in this manuscript:

AWGN	Additive White Gaussian Noise
BER	Bit Error Rate
CIR	Channel Impulse Response
CFO	Carrier Frequency Offsets
CFR	Channel Frequency Response
CP	Cyclic Prefix
CSS	Chirp Spread Spectrum
DFnT	Discrete Fresnel Transform
EDR	Effective Data Rate
FDE	Frequency Domain Equalization
FFT	Fast Fourier Transform
FEC	Forward Error Correcting
EFR	Errorless Frame Ratio
IDFnT	Inverse Discrete Fresnel Transform
IFFT	Inverse Fast Fourier Transform
ICI	Inter-Carrier Interference
ISI	Inter-Symbol Interference
LLR	Logarithm Likelihood Ratio
LS	Least Square
MMSE	Minimum Mean Square Error
MSE	Mean Square Error
OCDM	Orthogonal Chirp Division Multiplexing
OFDM	Orthogonal Frequency Division Multiplexing
PER	Packet Error Ratio
PN	Pseudo-Noise
PSK	Phase-shift Keying
QAM	Quadrature Amplitude Modulation
QPSK	Quaternary Phase Shift Keying
RMS	Root Mean Square
SNR	Signal-to-Noise Ratio
UWAC	Underwater Acoustic Communication
TDE	Time Domain Equalization
TVIR	Time-Varying Impulse Response
SISO	Single-Input Single-Output
SIMO	Single-Input Multiple-Output
SC	Single-Carrier
